# Physical activity—the past, present and potential future: a state-of-the-art review

**DOI:** 10.1093/heapro/daae175

**Published:** 2025-01-21

**Authors:** Matthew Mclaughlin, Peter McCue, Brittany Swelam, Joey Murphy, Sarah Edney

**Affiliations:** UWA Centre for Child Health Research, University of Western Australia, 35 Stirling Highway, Crawley, Western Australia, 6009,Australia; School of Medicine and Public Health, University of Newcastle, University Drive, Callaghan, New South Wales, 2308,Australia; Hunter Medical Research Institute, Lot 1, Kookaburra Crescent, New Lambton Heights 2305, New South Wales, Australia; Centre for Prevention, Implementation and Population Health, University of Newcastle, University Drive, Callaghan, New South Wales, 2308,Australia; School of Population Health, University of New South Wales, High Street, Kennington, New South Wales, 2052,Australia; Faculty of Health, School of Exercise & Nutrition Sciences, Deakin University, 221 Burwood Highway, Burwood, Victoria, 3125,Australia; School for Policy Studies, University of Bristol, Senate House, Tyndall Avenue, Bristol, BS81TH, UK; Saw Swee Hock School of Public Health, National University of Singapore, 12 Science Drive 2, Singapore, 117549,Singapore

**Keywords:** physical activity, advocacy, policy, interventions, systems, scale-up

## Abstract

This is a state-of-the-art review of historical developments, current approaches and recommended future directions in physical activity (PA) research, practice and policy. Since the early epidemiological studies in the 1950s, PA research has developed from within a biomedical paradigm. There is now a strong evidence base linking PA with positive health outcomes. PA is currently understood as a multi-sector issue, requiring a multi-sector solution (e.g. transport, urban design, sport), resulting in multiple individual and societal benefits (e.g. addresses multiple Sustainable Development Goals), however, there is a disconnect between interventions, policy and practice. This may be due to limited cross-sector collaboration between, and within, the public and private sectors. Furthermore, the mix of policy instruments employed by governments to implement PA policy to date has been dominated by soft (e.g. communication) rather than hard options (e.g. fiscal). To progress in PA promotion, we need to move beyond health outcome and intervention evidence generation (e.g. focus on testing efficacy in highly controlled settings), to more complex, real world, politically informed, multi-sector, scale-up and policies, while concurrently collecting data to evaluate such efforts (e.g. natural experiments and evaluations of the policy process). PA programs may benefit from greater incorporation of public policy considerations, so that proposed interventions and policies are designed with potential political constraints in mind. We conclude by providing a call to action to advance the understanding of the role of politics in PA, in order to develop politically informed action on PA.

**Contribution to Health Promotion**

- A brief history of the development of physical activity (PA) research, practice and policy—including a timeline of key milestones.
- A reflection on the current approaches to PA—including a summary of real-world PA case studies.
- Highlights the need to enhance our understanding of political insights, approaches and tools to help influence decisions, policymakers and politicians.
- As a group of early career researchers, we provide a call to action to advance the understanding of the role of politics in PA, in order to develop politically informed action on PA.

## INTRODUCTION

Research, practice and policy related to physical activity (PA) have gained considerable prominence (Blanchard *et al*., 2013; Shilton *et al*., 2013; Piggin, 2020b; Pogrmilovic *et al*., 2020; Lee *et al*., 2021) since the first epidemiology studies in the 1950s highlighting the health benefits of PA (Paffenbarger *et al*., 2001). Consequently, our understanding of PA, its benefits, the recommended quantity, and to some extent how to promote it, has evolved over time. Despite this, insufficient PA is increasing globally (Strain *et al*., 2024). Half of all countries and two-thirds of regions have increasing trends in insufficient PA prevalence, since 2000 (Strain *et al*., 2024). A change in the direction of the PA trajectory is urgently needed, if the global target of a 15% reduction in insufficient PA (from the 2010 baseline) is to be met by 2030 (World Health Organization, 2018b; Ding *et al*., 2024). Reflection is crucial for understanding current approaches to PA research, practice and policy; and identifying necessary next steps to alter the global PA trajectory. This article presents a non-exhaustive state-of-the-art review of historical developments since the 1950s, current approaches and recommended future directions in PA research, practice and policy.

## POSITIONALITY

The author team recognize that they all work in high-income countries (Australia, Singapore, the United Kingdom). The team has expertise in research, practice and policy in high-income countries across academia, not-for-profit advocacy groups and government. The team recognizes their collective experience and background is largely limited to English-speaking high-income countries, and this will have impacted, and limited, the lens from which this review has been conducted. The team recognize the importance of considering the inequalities and differences in contexts, including places, people and cultures, in PA research, practice and policy (Knuth *et al*., 2024).

## WHERE HAVE WE COME FROM: WHAT HAVE BEEN THE HISTORICAL DEVELOPMENTS IN PA?

Scholars have long-studied human movement, but it is challenging to suggest when PA research, practice and policy started, per se (Piggin, 2020b). This article uses the 1950s as a historical starting point of PA, based on the first epidemiological studies linking PA and health benefits (Varela *et al*., 2018). PA-related research emerged from several sectors during the first half of the 20th century (MacAuley, 1994; Paffenbarger *et al*., 2001). This included research, practice and policy in physical education, exercise physiology, active transport, workplace exertion and leisure time exercise (MacAuley, 1994; Berryman, 2010; Corbin, 2012). As such, this section is a non-exhaustive history of PA since the 1950s.

### Developing an understanding of PA through a biomedical lens

From within a biomedical science paradigm (using biomedical research methods), studies conducted in the 1950s began to show the positive health impacts of being physically active. This included studies that demonstrated the relationship between higher levels of PA and a reduced risk of coronary heart disease (Morris *et al*., 1953) and the relationship between running and cardiorespiratory fitness (Karvonen *et al*., 1957). The first published examples of evidence-based PA guidelines for physicians and clinicians were during the 1970s, with a focus on exercise to improve physical function and fitness (American Heart Association, 1972, 1975; American College of Sports Medicine, 1975). Towards the end of the 1970s, the American College of Sports Medicine published a landmark report that collated relevant evidence and highlighted recommendations for the type and amount of exercise needed to improve fitness, releasing one of the earliest published exercise recommendations intended for the general population (Haskell, 2019). Earlier PA guidelines may have existed, perhaps unpublished or published in languages other than English.

### Building a broader understanding of PA

During the 1980s and early 1990s, research demonstrated broader health benefits of PA including reduced risk of hypertension, osteoporosis, type II diabetes, and symptoms of stress and anxiety, as well as potential relationships in reducing other health-inhibiting behaviours (e.g. smoking) (Powell and Paffenbarger, 1985; Fletcher *et al*., 1992). Perhaps for the first time, recommendations for PA were also developed in the USA by an expert committee, convened to review the epidemiological, physiological and clinical evidence for PA and to establish and communicate the frequency, intensity and amount of PA needed ‘for health promotion and disease prevention’ (Pate *et al*., 1995). These PA guidelines moved away from defining a minimum bout length (e.g. 10 min) of PA and instead counted all movement, representing a shift to active living. Thereafter, several other higher-income countries adopted (epidemiology-) evidence-based PA guidelines [e.g. the UK in 1996 (Department of Health and Social Care, 2019)].

With a growing evidence base for PA, the early 2000s saw greater advocacy for the health benefits of PA. For example, an international report concluded there was strong evidence that PA is protective against colon, breast (post-menopause) and endometrium cancers (World Cancer Research Fund & American Institute for Cancer Research, 2007). This period also saw guidelines focus on specific population groups, including children and young people, older adults, pregnant and postpartum women and people with disabilities (Institute of Medicine, 2007). In 2008, for the first time, the ‘Physical Activity Guidelines for Americans’ included guidance on both aerobic and muscle-strengthening activities (US Department of Health and Human Services, 2008). Global guidelines followed in 2010 (World Health Organization, 2020). Surveillance of PA participation at national and international levels also emerged (Craig *et al*., 2003; Hallal *et al*., 2012), with the long-term intention of guiding evidence-informed targets, informing actions and advocacy, and instilling accountability (Hallal *et al*., 2012; Ramírez Varela *et al*., 2022)—such as those produced by the World Health Organization recently (World Health Organization, 2022).

Several key frameworks for PA emerged in the early 2000s. For example, the Behavioural Epidemiology Framework (Sallis *et al*., 2000) suggested a phased sequential approach from establishing links between behaviours (e.g. PA) and health (e.g. type 2 diabetes), to evaluating interventions, and enabling translation of research into policy (e.g. funded strategies, laws, regulations) and practice (e.g. allied health activities designed to help people increase their PA) (Sallis *et al*., 2000). In this way, the links between PA and health established from emerging evidence were subsequently used to shape intervention targets. Intervention targets were also framed through the wide use of the socio-ecological model that outlined multiple levels of influence on PA and recommended interventions targeting individuals, social environments, physical environments and policies to achieve population-level change (Sallis *et al*., 2006). These frameworks, coupled with the shift in focus to active living (e.g. accumulating PA throughout the day) and the limitations of individual-level interventions, led to the understanding that intersectoral collaboration was necessary to address physical inactivity (Sallis *et al*., 2006).

### Recognition of PA as a global and cross-sectoral problem and priority

Increased understanding of the health-related benefits of PA, and of the proportion of populations meeting (or not meeting) guideline recommendations, led to PA promotion being seen as a global priority. This evolved understanding, in conjunction with the recognition of the multiple levels of influence affecting population-level PA behaviour (Sallis *et al*., 2006), led to the development of cross-sectoral PA policy and advocacy approaches (Schmid *et al*., 2006; Shilton, 2008). Global PA strategies, recommendations and action plans emerged and demonstrated the shift in recognition of PA as a ‘global priority’. This included the publication of a global strategy (World Health Organization, 2004), a call for action (Bull *et al*., 2010), recommendations (World Health Organization, 2010) and the establishment of international organizations, such as the ‘Global Advocacy for Physical Activity’ (GAPA) [est. 2006; (Blanchard *et al*., 2013)] and the ‘International Society for Physical Activity and Health’ (ISPAH) (Shilton and Milton, 2024). Attempts were made to link PA advocacy with other priorities such as obesity prevention, sport, active transport and the environment (Shilton, 2008)—to achieve synergistic impacts. However, implementation of national PA policies was limited, as they tended to be poorly resourced (e.g. Bellew *et al*., 2008).

Global recognition of PA also became evident within the public health academic community with the release of the 2012, 2016 and 2021 Lancet Series (Lancet, 2012, 2016, 2021). These landmark series collated and summarized global PA evidence. On the back of the Lancet Series in 2012, the Global Observatory for Physical Activity (GoPA!) was established in 2014 to collate national PA surveillance, research and policy globally (Pratt *et al*., 2024), and to compare between countries and regions through GoPA!. Subsequently, the substantial inequalities both within and between countries in terms of meeting guideline recommendations and capacities to promote PA were elucidated (Ramírez Varela *et al*., 2022).

Work was done to identify the ‘investments that work’ for PA promotion, to help inform what PA promotion approaches should be developed, implemented and scaled up (International Society for Physical Activity and Health, 2011, 2021; Milton *et al*., 2021). These ‘investments that work’ affirmed the idea that there is no single isolated investment that can deliver sufficiently meaningful increases in PA at the population level (Milton *et al*., 2021). Rather, it suggested that cross-sectoral approaches can and should be utilized, with the recognition that PA is both an issue affected by multiple sectors and a solution for multiple problems across sectors. For example, PA was directly and indirectly linked to multiple Sustainable Development Goals, such as climate action and sustainable cities and communities (e.g. active transport, public spaces for recreation, etc.) (International Society for Physical Activity and Health, 2017; Salvo *et al*., 2021).

The strong biomedical evidence base, alongside the understanding of PA as a cross-sectoral problem, led to the publication of a Global Action Plan for Physical Activity 2018–2030 (GAPPA) (World Health Organization, 2018a), which included the global voluntary target for a 15% relative reduction in the global prevalence of physical inactivity in adults and adolescents by 2030 (World Health Organization, 2018a). The GAPPA 2018–2030 reaffirmed that increasing PA requires a systems-based approach—that there is no single-policy solution (Bull *et al*., 2020). However, recent work has demonstrated the challenges of advocating for the implementation of the plan across global regions (Murphy *et al*., 2022). Though examples of PA policy development across all levels of government have continued to expand, implementation of such policies remains limited and poorly resourced (Pratt *et al*., 2023). This is perhaps partly explained by the impacts of COVID-19 on PA policy globally (Richards *et al*., 2024).

Despite the obvious progress documented, recent surveillance data show that globally 81% of adolescents (Guthold *et al*., 2019) and 31% of adults (Strain *et al*., 2024) do not meet the PA guidelines. To achieve the GAPPA 2018–2030 targets (World Health Organization, 2018a), we must ask two important questions: how do we currently approach PA; and, what are the recommended future directions in PA research, practice and policy? Table 1 provides a brief historical timeline of key milestones in the development of PA as a field, since the 1950s.

**Table 1:** Key milestones in the development of PA research, practice and policy since the 1950s

	Milestone
	**Developing an understanding of PA through a biomedical lens**
1953	Relationship between Coronary Heart Disease (CHD) and occupational PA identified in London Bus Workers study (Morris *et al*., 1953).
1957	Relationship between cardiorespiratory fitness and running identified (Karvonen *et al*., 1957).
1967	Physical inactivity determined to increase the risk for heart disease in Framingham Heart Study (Kannel, 1967).
1975	Longitudinal study, San Francisco Longshoremen Study, identifies differences in coronary heart mortality based on strenuous, moderate and low activity outputs (Paffenbarger and Hale, 1975).
1972–1975	Early exercise recommendations published by professional organizations serving as clinical exercise guidance for clinicians such as general practitioners, exercise scientists and exercise rehabilitation clinicians (American Heart Association, 1972, 1975; American College of Sports Medicine, 1975).
1972–1978	Some of the earliest documented evidence-based exercise guidelines intended for general public produced by ACSM (Haskell, 2019).
1979	Expanded health benefits of PA identified in Framingham Heart Study (Kannel and Sorlie, 1979).
	**Building a broader understanding of PA**
1985	Widely adopted definitions of PA, exercise and physical fitness (as distinct entities) developed (Caspersen *et al*., 1985).
1985–1992	Broader health benefits of PA including (Powell and Paffenbarger, 1985) reduced risk of hypertension, osteoporosis, type II diabetes, and symptoms of stress and anxiety, as well as potential relationships in reducing other health-inhibiting behaviours (e.g. smoking) (Fletcher *et al*., 1992) identified. Inverse and causal relationship between PA and CHD deduced from published review of epidemiological evidence (Powell *et al*., 1987).
	**Recognition of PA as a priority**
1992–1998	Early PA advocacy/policy publications released in the USA, Canada, greater Europe, Great Britain, Finland and Australia (Pogrmilovic *et al.*, 2018; Varela *et al.*, 2018)
1992	Early surveillance of PA participation at the national level emerged in the UK (Sports Council Health Education Authority, 1992) and other developed countries (Bellew *et al*., 2008).
1995–1996	US population-level recommendations developed and released by the Centers for Disease Control and American College of Sports Medicine (Pate *et al*., 1995), followed by several other organizations shortly thereafter (e.g. WHO/FIMS Committee on Physical Activity for Health, 1995; National Institute Health (NIH), 1996). Centers for Disease Control (USA) report *‘Physical Activity and Health: A Report of the Surgeon General’* emphasizes importance of moderate level of activity for health benefits and that regular PA is related to the volume of health benefits (U.S. Department of Health and Human Services, 1996)
1997	Global surveillance of PA discussed at World Health Organization (WHO) meeting for the *International Physical Activity Questionnaire (IPAQ)*, resulting in subsequent testing of IPAQ and dissemination (Craig *et al*., 2003; Hallal *et al*., 2012)
1998–2006	Behavioural epidemiology framework suggesting pathway for PA and health studies leading to evidence-based interventions released; recognition of influences on PA participation (Sallis and Owen, 1998) and subsequent release of proposed ecological framework for improving PA (Sallis *et al*., 2006)
2007	Focus of guidelines placed on specific population groups, including children and young people, older adults, pregnant and postpartum women and people with disabilities (Institute of Medicine, 2007)
2007	Cancer and physical activity were linked by the Second Expert Report (World Cancer Research Fund & American Institute for Cancer Research, 2007). Specifically, the report concluded there was strong evidence that physical activity is protective against cancers of the colon, breast (post-menopause) and endometrium.
	**Recognition of PA as a global and cross-sectoral problem and priority**
2000–2014	Regional PA advocacy networks established: Red Actividad Fisica de las Americas/Physical Activity Network of the Americas (RAFA/PANA; 2000); European Network for Promotion of Health-Enhancing Physical Activity (HEPA Europe; 2005); Asia Pacific Physical Activity Network (AP-PAN; 2005); Global Advocacy for Physical Activity for Physical Activity (GAPA; 2007; later merged with the International Society for Physical Activity and Health); Africa Physical Activity Network (AFRO-PAN; 2010); Global Observatory for Physical Activity (GoPA!; 2012); Active Healthy Kids Global Alliance (AHKGA; 2014) (Blanchard *et al*., 2013)
2001–2006	Studies to explore the relationship between PA and policy (individual, social and environmental perspectives) (Brownson *et al*., 2001; Saelens *et al*., 2003; Sallis *et al*., 2006)
2002–2004	WHO and Centers for Disease Control and Prevention (CDC) consultations on development of PA policies and resultant launch of the first global policy document *‘Global Strategy on Diet, Physical Activity and Health’* (2004) (World Health Organization, 2004)
2004	PA policy defined in publication (Bull *et al*., 2004)
2005–2012	Early emergence of PA policy branches in literature: built environment and global PA surveillance (Varela *et al*., 2018)
2006	First dedicated International Congress on Physical Activity and Public Health (ICPAPH)
2008	Formation of the *International Society for Physical Activity and Health (ISPAH)*
2010–2011	*‘Toronto Charter for Physical Activity: A Global Call for Action’* launched at the third ICPAPH, later translated into 23 languages (2010–2011) (Bull *et al*., 2010)
2010	Launch of first global recommendations for PA by the WHO, *‘Global Recommendations on Physical Activity for Health’*
2010	Physical inactivity identified as the fourth leading risk factor for NCDs in the WHO *‘Global Burden of Disease’* report
2011–2012	Physical inactivity acknowledged as key risk factor for non-communicable diseases (NCD) at United Nations (UN) meeting, later resulting in the release of *‘NCD Prevention: Investments that Work for Physical Activity’*; translated into seven languages (International Society for Physical Activity and Health, 2011)
2012	First ‘*Lancet Physical Activity Series’* published, identifying physical inactivity as a pandemic, and inclusive of global perspectives on steps to address physical inactivity (Hallal *et al*., 2012; Kohl *et al*., 2012; Lee *et al*., 2012); subsequent Lancet Physical Activity Series released in 2016 and 2021 (Lancet, 2016, 2021)
2012–2013	GAPA and other regional PA networks successfully advocate for the inclusion of a global target (10% relative reduction in physical inactivity) and indicator on physical inactivity in the WHO Global NCD framework, *‘Global Action Plan for the Prevention and Control of Noncommunicable Diseases 2013-2020’* via two key position statements (World Health Organization, 2012; Blanchard *et al*., 2013)
2012	Global target of reducing physical inactivity by 10% by 2025 endorsed by the WHO General Assembly
2014	The Global Observatory for Physical Activity (GoPA!) was established in 2014 to collate national PA surveillance, research and policy globally. The first set of global country cards was published in 2015 (Pratt *et al.*, 2024).
2016	Published Lancet review *‘Progress in over the Olympic Quadrennium’* highlights three key barriers to physical activity policy implementation: (1) insufficient workforce to implement PA policies; (2) the lack of formation of multi-sectoral partnerships in relevant sectors (transport, education, sport, recreation, urban planning) and (3) absence of clarity on likely feasible/effective actions for physical activity in given context (Sallis *et al*., 2016)
2016	ISPAH publishes *‘The Bangkok Declaration on Physical Activity for Global Health and Sustainable Development’*, linking physical activity and several sustainable development goals for the first time (International Society for Physical Activity and Health, 2017)
2018	*‘Global Action Plan on Physical Activity 2018-2030’* (GAPPA) released by the WHO; global target of reducing physical inactivity by 15% by 2030 (World Health Organization, 2018)
2018–2019	Pooled data set of 1.9 million adults from 168 countries identifies 27.5% of adults as insufficiently active in 2016, with no improvements in physical inactivity identified between 2001 and 2016; Pooled data set of 1.6 million adolescents from 146 countries identifies 81.0% of adolescents (aged 11–17 years) as insufficiently active in 2016, with some improvements in physical inactivity identified between 2001 and 2016 in boys, but not girls (Guthold *et al*., 2018, 2019)
2020	*‘8 Investments That Work for Physical Activity’* released with associated advocacy toolkit by ISPAH (International Society for Physical Activity and Health, 2021; Milton *et al*., 2021)
2022	WHO PA recommendations expanded to include pregnant people and people with disabilities (Bull *et al*., 2020)
2021	Opportunistic synergies between PA promotion and the UN sustainable development goals (SDG; *2030 Agenda for Sustainable Development*) identified in scoping review by physical activity experts and scientific literature, highlighting that at-scale physical activity promotion may also impact on the 15/17 SDGs (Salvo *et al*., 2021)
2021	WHO releases toolkits to support Global Action Plan on Physical Activity (2018–2030) and an advocacy brief, *‘Fair Play: Building a Strong Physical Activity System for More Active People’*, advocating for countries to rebuild stronger and fairer PA systems following the COVID-19 pandemic (World Health Organization, 2021a, 2021b, 2021c, 2021d, 2023)
2023	The Global Alliance for the Promotion of Physical Activity publishes consensus statement *‘Hamburg Declaration’* with five key messages: (1) Promote PA as medicine; (2) Lobbying decision-makers; (3) Adapt PA to the individual, community, and their surroundings; (4) Leverage the latest tech; (5) Call for more trials on effectiveness and implementation of policies and programs (Steinacker *et al*., 2023)
2022–2023	Policy evaluation in PA evolves through dedicated networks (e.g. system-based Policy Evaluation Network (Kamphuis *et al*., 2022)) and PA policy benchmarking framework ‘MOVING’ developed and applied in 27 EU countries to assess and monitor national progress on government PA action (Vlad *et al*., 2023)

This table was informed by prior research (Blanchard *et al*., 2013; Shilton *et al*., 2013; Pogrmilovic *et al*., 2018; Varela *et al*., 2018).

## WHERE ARE WE NOW: HOW DO WE CURRENTLY APPROACH PA?

The section discusses the dominant types of PA evidence. It also discusses more recent developments in the application of systems science and the evaluation of PA policy implementation globally.

### Generating relevant PA intervention evidence

Our current approach to evidence generation for PA interventions is ‘innovation’ and ‘evidence-based’, largely driven by the traditional biomedical paradigm where the gold-standard approach is testing an innovation for efficacy (e.g. drug) using a randomized controlled trial (often referred to as the evidence-based ‘gold standard’) (Grossman and Mackenzie, 2005). Most research on PA interventions has been at this early end of the Behavioural Epidemiology Framework (Sallis *et al*., 2000; Lee *et al*., 2021), this has perhaps been to the detriment of practice-driven solutions and the acknowledgement of varied contexts, environments and cultures. Practice-driven solutions (i.e. developed outside academia) are often developed under dynamic real-world conditions, unfortunately often with limited evaluation (Crane *et al*., 2020; David *et al*., 2020).

Efficacy and effectiveness evidence generation has primarily occurred in the context of high-income countries, where a minority of the global population lives, triggering concern regarding the adaptability of these interventions to low- and middle-income countries (Ramírez Varela *et al*., 2022). Even when moving from small, highly controlled research settings to larger, and more real world, settings it has been a challenge for interventions to retain the effectiveness of their interventions—typically experiencing a ‘voltage drop/ scale-up penalty’ in effectiveness (Lane *et al*., 2021). Therefore, the evidence base for PA interventions is driven largely by efficacy studies, with unclear pathways to impact (Rütten *et al*., 2016; Wolfenden *et al*., 2016; Lee *et al*., 2021). This might be explained by the academic system (including grant schemes) assigning greater importance to ‘efficacy’ (and to some extent ‘effectiveness’) as the key outcomes of evaluations, rather than cost or political satiability, for example.

Decision-makers enacting policy may be more motivated by outcomes other than effectiveness (e.g. cost, feasibility, political satiability) (Milat *et al*., 2019) and may not make policy decisions based on evidence alone (Fafard, 2015; Cairney and Oliver, 2017; Fafard *et al*., 2022). For example, policymakers and politicians may prefer certain types of evidence to others (Wolfenden *et al*., 2024), or have a preference for softer (e.g. communication) rather than harder (e.g. infrastructure, taxes) policy actions (de Leeuw, 2023). Additionally, it is possible that the stated preference for a certain type of evidence (Wolfenden *et al*., 2024) may mismatch with the actual evidence used in policy-making. Taken together, this perhaps explains why few PA trials with proven efficacy have moved along the ‘pipeline’ from efficacy to dissemination on a larger scale (Wolfenden *et al*., 2016; Lee *et al*., 2021). Despite these weaknesses of the current evidence base, there are now many interventions that have demonstrated their effectiveness (in particular contexts), as well as practice-driven solutions to increase PA. In Table 2, we have summarized a few examples of these case studies, as a way to understand our current evidence base of effective interventions.

**Table 2:** Example case studies of PA interventions and policies^*^

Investment area	Case study example	Country	What it did?
Active travel	Paris area wide 30 km/h speed limit (British Broadcasting Centre, 2021)	France	Reduction of area-wide speed limits across Paris to 30 km/h to improve road safety and sharing of public space for active and soft mobility. In addition, reduction in street parking bays, and re-design of streets to increase cycling and walking provision.
	Car Free Day (Lagos Government, 2022)	Nigeria	Closure of roads to prevent vehicular access in city of Lagos (population ~20 million) to encourage of uptake of active non-motorized transport modes instead (e.g. cycling and walking) Community activities (e.g. cycling training, competitions, skating, stretching, music and dance) in public spaces
	Opportunistic pop-up separated cycleway construction during COVID-19 (Harris and McCue, 2022)	Australia	Examination of policy development process that led to the rapid installation of pop-up cycleways in Sydney (Australia) in response to COVID-19 Uses opportunistic pop-up cycleway construction to provide insights for cycling policy development in jurisdictions traditionally burdened by skepticism and reluctance to implement bicycle infrastructure
Active urban design	Barcelona ‘superblocks’ (Amati *et al.*, 2024)	Spain	Restriction of vehicle traffic (banned or restricted to safe speeds < 30 km/h) in approx. 400 m × 400 m city blocks to prioritize/promote walking and cycling on ‘green streets’ Return of open space previously utilized for private vehicle parking, creating a vibrant public space for walking, cycling and living
	Our Voice (East Palo Alto, NEAAT program) (Buman *et al*., 2012)	United States of America	Citizen science approach to improving local environment for active living, using Discovery Tool app, and four-step Our Voice process: discover, discuss, activate and change Enabled senior residents to assess and address factors impeding walkability in local neighbourhood via advocacy and skills training Formation of resident-based community advocacy team resulted in relationship with city planning department, implementation of sidewalk inventory and repair program and allocation of $1 million from the city planning department for a safer public health environment plan Our Voice approach adapted and utilized across numerous projects in 17 countries
Healthcare	Green Prescription (GRx) (Tewhatuora Government of New Zealand, 2024)	New Zealand	Prescription by healthcare professional to support patient in becoming more physically active Ongoing support for patients: monthly telephone calls, face-to-face meetings and/or group support in a community setting
	Moving Healthcare Professionals (Sport England, 2024)	United Kingdom (England)	Evidence-based training modules and resources to support healthcare professionals in PA promotion and prescription Embedded into medical curricula
Public education/mass media	This Girl Can (This Girl Can, 2024)	United Kingdom	Mass media campaign across social media, television, print media, and billboards to address the activity gap in women and girls Additional subsequent campaigns launched that focused on women aged 40–60 (Phenomenal Women) and from different backgrounds (Fit Got Real) Aimed to increase representation and relatability of exercise and PA to women Provided accessible resources to be physically active
	Bike Is Best (UK) (BikeIsBest, 2024)	United Kingdom	Mass media campaign across social media, television, print media and billboards advocating for the use of bicycles for journeys under five miles in lieu of an automobile Provided research and educational resources about the impact of bicycles/reducing automobile usage on the environment, health outcomes, bicycle safety, etc.
Sport and recreation for all	Active Kids Sport Vouchers (New South Wales) (Foley *et al*., 2021)	Australia	Government-funded $50 voucher for parents/guardians, of school-aged children to assist in covering costs of registration and membership fees of sport and/or active recreation. Evaluation of the program suggested that children met the PA guidelines for one additional day per week after uptake of the voucher, and that 42.4% of total structured activity time (outside of school) was attributable to the voucher-specific activity Similar voucher schemes exist in most Australian jurisdictions, for example, KidSport in Western Australia provides low-income families $300 for sports club fees, club uniforms or club equipment for children (Department of Local Government Sport and Cultural Industries, 2024)
	National Steps Challenge (Ang *et al*., 2022)	Singapore	National Steps Challenge provides free fitness trackers to Singaporean adults that accumulate rewards based on daily step and MVPA counts
Workplaces	Workplace Challenge (Adams *et al*., 2018)	United Kingdom (England)	Program delivered nationally, targeting inactive employees via workplaces, consisting of four core components: (1) activity logs for recording sport, PA and active travel; (2) national activity logging challenges; (3) local activities/challenges and (4) champions to assist with embedding PA into culture of workplace Led and managed by national and local sporting bodies, as well as additional partners At follow-up, 86% of the previously inactive participants were meeting the recommended MVPA guidelines
	Step Count Challenge (Step Count Challenge, 2024)	United Kingdom (Scotland)	Workplace team-based step challenge encouraging walking to work, walking meetings, and lunchtime walks Team step tracking completed via apps; additional tips, motivation and prizes available Promotion packs provided for participants to recruit additional colleagues
Community programmes	Ciclovía (World Economic Forum, 2024)	Colombia	Weekly closure of multiple highways and streets to vehicle traffic to foster active recreation, active modes of transport, and community engagement Community exercise and dance classes, musical performances, health screenings, food vendors and shops included along the closed roads to encourage healthy lifestyles Modern-day Ciclovía in Bogotá is a multi-sectoral collaboration that sees 1.4 million participants every Sunday and spans 127 km, with 85% of participants meeting recommended PA guidelines (Mejia-Arbelaez *et al*., 2021). Estimated 497 Ciclovía-style programs have occurred across 27 countries globally ( Mejia-Arbelaez *et al*., 2021).
	Parkrun (Parkrun, 2024)	Global	Volunteer-led 5 and 2 km events occurring weekly in open community spaces, originated in the UK in the early 2000s, and designed to encourage a sense of community and movement for all ages and abilities Has since expanded to 22 countries
Whole-of-school programmes	Transform-Us! (Salmon *et al.*, 2022)	Australia	School-based program that focuses on increasing PA, decreasing sedentary behaviour and improving other health and education outcomes via active academic lessons, active breaks, health and wellbeing classes, active environments and family engagement Provides an online training platform for teachers, principals and schools to substitute sedentary lessons for physically active lessons that reimagine the learning process and encourage transformation of active environments, including the classroom and home
	Creating Active Schools Improvement Tool (Chalkley *et al.*, 2024)	United Kingdom	Professional development program designed to support schools in identifying target improvement areas and existing strengths to build upon for a healthier and more physically active school Stemmed from the Creating Active Schools framework developed to identify components necessary for implementing a whole-of-school PA program
Other	Active Bradford (Active Bradford, 2024)	United Kingdom	Partnership of organizations that aim to develop and deliver active opportunities using a systems approach Plan and develop strategic plans and programs, advocate for the role of PA and sport in broader strategic plans and influence decision-makers, and promote active opportunities in campaigns and other communication mediums Nine key priorities for action: (1) active schools, children and young people; (2) active neighbourhoods and communities; (3) sport and active recreation; (4) partnering with health anchor organizations; (5) workplaces; (6) community access to parks and open spaces; (7) built environment: healthy/active homes and environments; (8) active travel and (9) working with partners to deliver strong message

^*^Example case studies are largely drawn from high-income countries, which is a limitation of the examples provided.

### Moving from interventions to effective policy

A PA intervention can be defined as ‘any form of PA service, programme or strategy aimed at increasing PA levels with general or special populations’ (Murphy *et al*., 2023, p. 2). The term policy tends to be used as an umbrella term for laws, regulations, strategic documents/action plans and political (i.e. governmental) structures and processes. For the purpose of this article, we define PA policy as ‘…agendas, structures, funding and processes that affect development, implementation or adaptation of physical activity interventions’ (Rütten *et al*., 2016, p. 554). In this way, PA policies can also be considered as a form of PA intervention too—as they are not mutually exclusive from interventions. However, PA policies can differ from PA interventions, as PA policies can both increase levels of PA themselves and also support or impede other PA interventions (Gelius *et al*., 2020). As such, policies can be viewed as the parameters for a package of interventions (de Leeuw, 2023).

The evidence base demonstrating the efficacy of specific PA interventions is larger than the evidence base for PA policy development, content and implementation (Rütten *et al*., 2016; Gelius *et al*., 2020). PA policy research to date has predominately focused on the monitoring or components of policies (Pogrmilovic *et al.*, 2018). Less attention has focused on the PA policy development process, to interrogate why recommended evidence-based components have (or have not) been included, with limited exceptions (Noël Racine *et al*., 2022; Malcolm *et al*., 2023).

Given the compelling nature of the evidence of what is required to increase population levels of PA and what the benefits of doing so would be, public health researchers have questioned why there is not more widespread implementation of policies to support such increases (Pratt *et al.*, 2015). To be implemented, policy needs to be operationalized via policy instruments (de Leeuw, 2007). Policy instruments include (Althaus *et al*., 2020; Gelius *et al*., 2021b):

- Regulatory instruments (law, rule, regulation, directive, standard, sanction)
- Economic/fiscal instruments (fiscal, tax, fee, subsidy, price, fine, spend on services)
- Soft instruments (campaign, code of conduct, recommendation, voluntary agreement, partnership and coordination)
- Advocacy (arguing a case)
- Framing/narrative (using storytelling to frame issues and solutions)

A recent analysis of the World Health Organization’s (WHO) policies attempting to address physical inactivity (Gelius *et al*., 2021) found that ‘soft’ instruments (e.g. communication, recommendations such as global strategy and call to action and networking) dominate current PA policy implementation (Gelius *et al*., 2021). On the contrary, ‘hard’ instruments (e.g. regulatory and economic/tax policy) had been used frequently to address other health behaviours, such as tobacco and alcohol reduction (Gelius *et al*., 2021b), but less frequently in PA (Nau *et al*., 2021). This may partly explain why PA has been under-recognized in policy attention (Bull and Bauman, 2011), whilst other health behaviours (e.g. nutrition, tobacco) have received greater political attention and thus, harder policy instruments (e.g. laws, taxes).

A scoping review of global studies analysing indicators, development and content of national-level PA policies found that 168 countries had prepared PA policies, however, policy evaluation was lower in low- and middle-income countries (Pogrmilovic *et al.*, 2018). Much of this policy evaluation and monitoring was done through the GoPA! (Ramirez Varela *et al*., 2021). However, despite the large global coverage of policy monitoring, there is a need for improved conceptualization of policy, greater reliance on existing theoretical frameworks, and the use and further development of standardized methods for policy analysis (Pogrmilovic *et al.*, 2018). Attempts to measure policies across countries are increasing and include groups such as the Policy Evaluation Network (Kamphuis *et al*., 2022) and GoPA! and Global Observatory for Physical Education (GoPE!) (Ramirez Varela *et al*., 2021; Martins *et al*., 2023). A number of tools now exist to assess the development and implementation of PA policies and this is a rapidly developing field within PA (Bull *et al*., 2015; Pogrmilovic *et al*., 2019; Aubert *et al*., 2022; Kamphuis *et al*., 2022; Oldridge-Turner *et al*., 2022; Resendiz *et al*., 2024).

### Systems approaches to PA

The Global Action Plan on Physical Activity 2018–2030 proposed that effective action on PA requires an integrated, system-wide approach in consultation with policymakers and stakeholders from multiple sectors (World Health Organization, 2018a). However, despite the widely acknowledged need for systems approaches, this has been a challenge for PA. Operationalizing what this means for policy practice is therefore of critical importance (Gelius *et al*., 2021; de Leeuw, 2021). Case studies and evidence of systems approaches have started to emerge (Murphy *et al*., 2021; Nau *et al*., 2022; Nobles *et al*., 2022), including the formation of professional development and networking groups (Systems Evaluation Network, 2022).

One particular challenge to systems approaches to PA has been cross-sector cooperation (Hämäläinen *et al*., 2016). Identifying what level/department of government has responsibility for PA-related infrastructure, policy development and programs has been a challenge (Merom *et al*., 2010; McCue, 2017), but has mainly been led by sport and/or health sectors (Gelius *et al*., 2021a). For example, walking promotion in England was packaged as either a health, transport or an environment issue—which in turn impacted upon which agency within the government took responsibility for delivery (Milton and Grix, 2015). In such cases, the challenge also becomes about who is financially responsible for funding such programs.

More practical examples and evaluations are required to build an evidence base of how to implement systems approaches to PA (Murphy *et al*., 2021; Nau *et al*., 2022; Nobles *et al*., 2022). This will also warrant building an understanding of how costs are distributed across sectors, as well as cost-benefit ratios (Abu-Omar *et al*., 2017).

Taken together, the current challenges in PA include generating relevant evidence, moving from interventions to effective policy, and operationalizing a systems approach. To address these challenges, the next section provides a call to action and suggests future directions in PA research, practice and policy.

## WHERE TO NEXT: WHAT ARE THE RECOMMENDED FUTURE DIRECTIONS IN PA RESEARCH, PRACTICE AND POLICY?

**Fig. 1:**
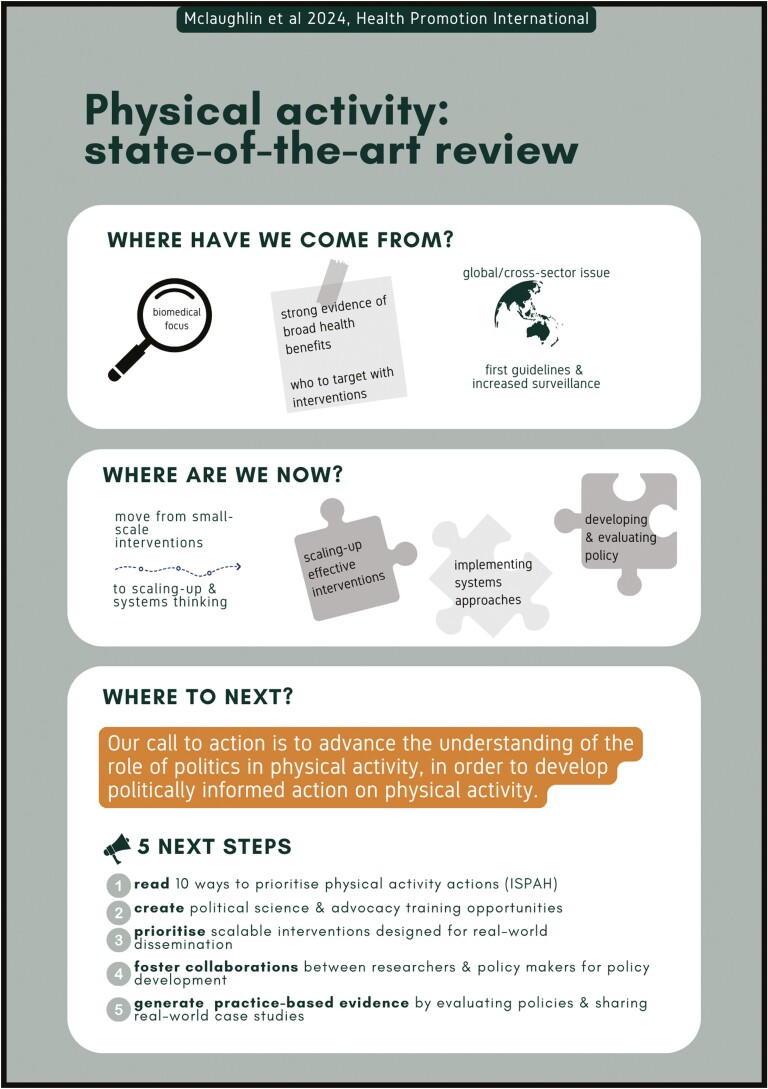
Visual abstract and call to action to advance the role of politics in PA.

Our call to action, as a group of early career researchers, is to advance the understanding of the role of politics in PA, in order to develop politically informed action on PA (Figure 1). This is especially important in the post-pandemic era, as COVID-19 widely disrupted PA policy implementation (Richards *et al*., 2024). Whilst there have been some calls to get ‘politics’ out of evidence-based public health policy, our view is that politics is omnipresent. Consequently, we should seek to understand it, so that we can influence it (Piggin, 2020a; Fafard *et al*., 2022). This will inevitably involve drawing on research from adjacent fields, such as sociology and political science (Piggin *et al*., 2020) (Masnfield, 2017; Kay, 2017). We affirm the need to move beyond a biomedical framework of evidence generation, and instead, focus on the multi-sectoral and political collaboration necessary for PA and health in various contexts. As such, there is an opportunity for political choices to influence PA policy formation and implementation (Fafard, 2015). For research findings to inform efficient policy and implementation, studies need to move on from the ‘what’ to the ‘how’ (Brownson and Jones, 2009). Generating economic evaluation (e.g. cost, cost-effectiveness) evidence for different PA approaches will also be key for taking a more political approach to PA (Abu-Omar *et al*., 2017).

A political science approach could help analyse the process of policy change over time and not just the current content of PA policies (de Leeuw *et al*., 2014). It is pleasing to see a growing body of literature on the politics of PA, including critical discussions about the politics involved in PA advocacy, policy and practice (Piggin *et al*., 2024). A greater focus on the politics of PA could, for example, explore how policies are established and for whom (Malcolm *et al*., 2023; Piggin *et al*., 2024) and examine how decisions are made about which physical activities are funded and promoted (Piggin *et al*., 2024).

To develop evidence in the real world, researchers should increase their focus on the evaluation and knowledge translation of practice-driven interventions and policy actions—this will likely involve greater use of natural experiments and less reliance on controlled research designs (Crane *et al*., 2020; David *et al*., 2020). The key outcomes of these evaluations should be driven in partnership with stakeholders/key partners, and will likely necessitate the expansion of the narrow effectiveness/efficacy outcome focus of current intervention evaluations. This may subsequently enable the inclusion of policy and process outcomes relevant to a business case for a PA policy solution (Milat *et al*., 2019; Hoefer, 2022; International Society for Physical Activity and Health, 2022) such as:

- framing of the problem and solution, how they are perceived and how they align to complementary agendas;
- technical feasibility of the intervention, expertise, resources, delivery modes and infrastructure required;
- acceptability and support;
- costs of development, implementation and maintenance;
- sustainability;
- transferability across contexts and countries;
- potential reach and equity across populations.

Public health researchers, advocates and policymakers should be aware of the political nature of their work and of the methods and tools from political science that can help them build capacity for doing this (de Leeuw *et al*., 2021).

We provide five suggested next steps for PA professionals:

1. Read the ‘10 Ways to Prioritise Physical Activity Actions’ document (International Society for Physical Activity and Health, 2022) [and seek out other more detailed reading on the politics of PA, e.g. (Piggin *et al*., 2017; Piggin, 2020a)];
2. To only ‘pilot’ test PA interventions that are designed from the outset for the scale and context of the real world;
3. Create political science and advocacy training for PA professionals;
4. For researchers and policymakers to work together on policy instrument development, going beyond intervention development;
5. To bolster practice-based evidence by evaluating the impact of PA policy instruments and to share real-world case studies.

As a group of early career researchers, we dream of a world in which every single person can engage in health-enhancing PA throughout their day—a lofty dream. Current approaches are moving us in the right direction to achieve it, but we are concerned that at our current pace, it won’t happen soon enough—certainly not before we retire. We are not that patient, we already know that the benefits of a physically active world go far beyond health—and we acknowledge that we now need to enhance our understanding of political insights, approaches and tools to help influence policy design, decisions, policymakers and politicians.
